# Role of Cardiac A_2A_ Receptors Under Normal and Pathophysiological Conditions

**DOI:** 10.3389/fphar.2020.627838

**Published:** 2021-01-26

**Authors:** P. Boknik, J. Eskandar, B. Hofmann, N. Zimmermann, J. Neumann, U. Gergs

**Affiliations:** ^1^ Institut für Pharmakologie und Toxikologie, Medizinische Fakultät, Westfälische Wilhelms-Universität, Münster, Germany; ^2^ Cardiac Surgery, Medizinische Fakultät, Martin-Luther-Universität Halle-Wittenberg, Halle, Germany; ^3^ Bundesinstitut für Arzneimittel und Medizinprodukte, Bonn, Germany; ^4^ Institut für Pharmakologie und Toxikologie, Medizinische Fakultät, Martin-Luther-Universität Halle-Wittenberg, Halle, Germany

**Keywords:** A_2A_-adenosine receptor, contractility, ischemia, reperfusion, arrhythmias

## Abstract

This review presents an overview of cardiac A_2A_-adenosine receptors The localization of A_2A_-AR in the various cell types that encompass the heart and the role they play in force regulation in various mammalian species are depicted. The putative signal transduction systems of A_2A_-AR in cells in the living heart, as well as the known interactions of A_2A_-AR with membrane-bound receptors, will be addressed. The possible role that the receptors play in some relevant cardiac pathologies, such as persistent or transient ischemia, hypoxia, sepsis, hypertension, cardiac hypertrophy, and arrhythmias, will be reviewed. Moreover, the cardiac utility of A_2A_-AR as therapeutic targets for agonistic and antagonistic drugs will be discussed. Gaps in our knowledge about the cardiac function of A_2A_-AR and future research needs will be identified and formulated.

## Introduction

There have been many reviews on adenosine receptors (AR), specifically A_2A_-AR (Fredholm et al., 2001; Haskó and Pacher 2008; Fredholmet al., 2011; McIntosh and Lasley, 2012; Chen et al., 2013; Headrick et al., 2013; Burnstock and Boeynaems, 2014; Burnstock, 2015; Boros et al., 2016). However, there are few reviews on cardiac A_2A_-AR. The present work attempts to close this gap in the literature.

In their pioneering work on the pharmacology of adenosine in the heart, Drury and Szent-Györgyi (1929) showed that it can reduce the force of contraction and induce arrhythmias, namely bradycardia. Adenosine alone has a negative chronotropic effect on the sinus node, a negative dromotropic effect on the atrioventricular (AV) node, and a negative inotropic effect on atrial tissue; after β-adrenergic stimulation, adenosine has a negative inotropic effect on the ventricular tissue of most mammalian hearts (Shryock and Belardinelli, 1997). Receptors that are activated by adenosine are called P1 receptors and are differentiated from P2 receptors, which are preferentially activated by adenosine triphosphate (ATP); this agonist selectivity can be lost if high concentrations of ATP or adenosine are used.

The focus of the present review is the P1 receptors. There are four different receptors: A_1_, A_2A,_ A_2B_, and A_3_. In general, A_1_-AR and A_3_-AR inhibit adenylyl cyclase, while A_2A_-AR and A_2B_-AR stimulate adenylyl cyclase activities in the heart (Olsson and Pearson, 1990).

### Receptor Structure

**Figure 1:**
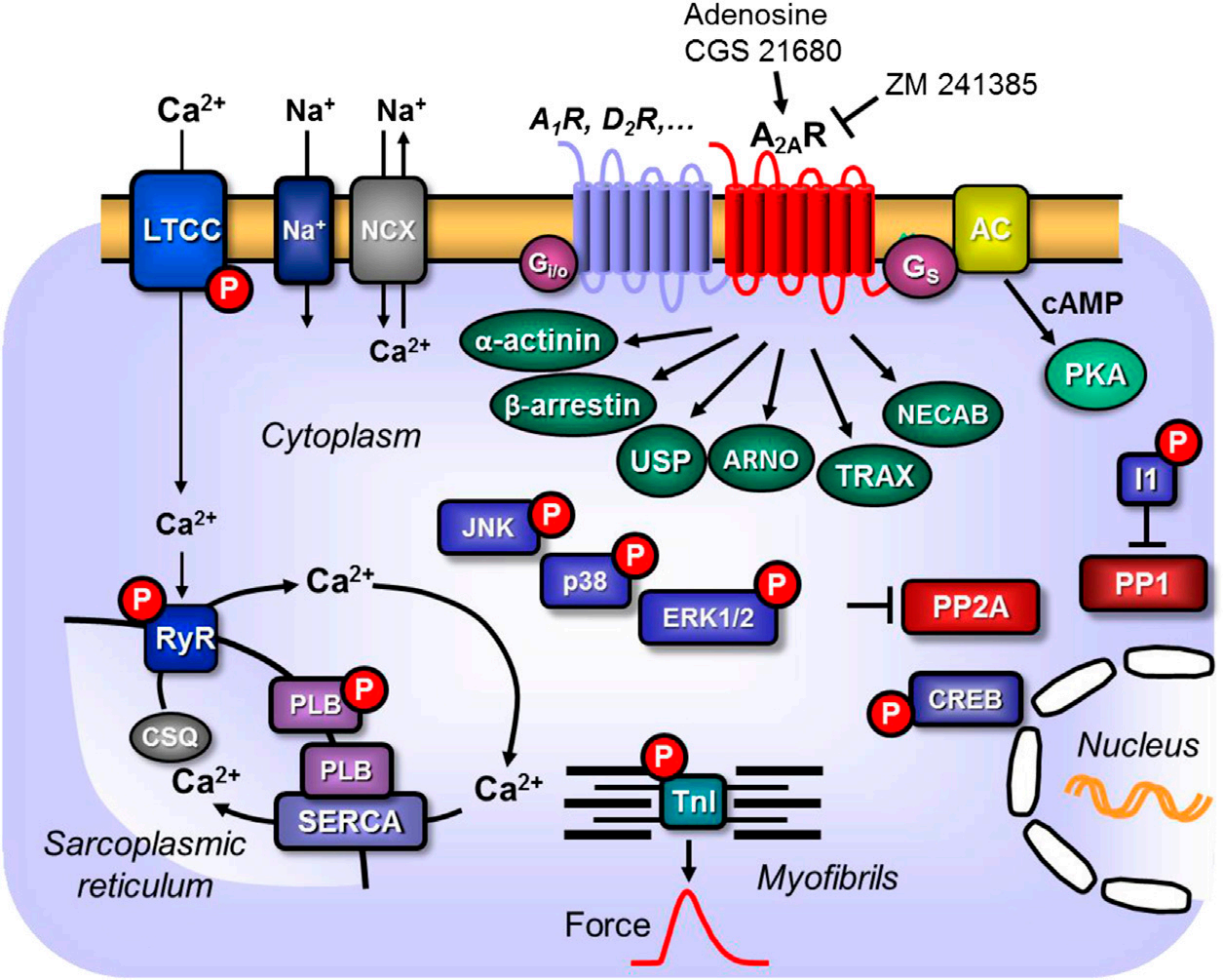
Scheme: Putative mechanism(s) of signal transduction of cardiac A_2A_-adenosine receptors (A_2a_-ARs). A_2a_-ARs via stimulatory G-proteins (Gs) can activate adenylyl cyclase (AC) which would enhance the 3′-5′cyclic adenosine-phosphate (cAMP)-levels in compartments of the cardiomyocyte and activate cAMP-dependent protein kinases (PKA) which would increase the phosphorylation state and thereby the activity of various regulatory proteins in the cell. Moreover, phosphorylation state and thus the activity of ERK1/2, JNK, p38 and CREB could be enhanced by pathways via arrestins. PKA-stimulated phosphorylation might also increase the current through the L-type Ca^2+^ channel (LTCC) and/or release of Ca^2+^ from the sarcoplasmic reticulum (SR) via the cardiac ryanodine receptor (RYR2); both processes would increase force of contraction by increasing the Ca^2+^ acting on myofilaments. In diastole, Ca^2+^ is pumped via the SR-Ca^2+^ ATPase (SERCA) from the cytosol into the SR. Activity of SERCA is increased by phosphorylation of phospholamban (PLB). The latter effect might also follow from inhibition of PP2A (a serine/threonine phosphatase: PP) activity by MAP kinases and subsequent increased phosphorylation state and thus activation of I-1 (a specific inhibitory protein of PP1) which will lead to decreased activity of PP1. Reduced activity of PP2A (and/or PP1) can increase phosphorylation of additional proteins and might thus increase the Ca^2+^ -sensitivity of myofilaments by dephosphorylation of the myosin light chains in the myofilaments which would increase force of contraction. Thus, A_2A_-ARs might increase the Ca^2+^ -sensitivity of myofilaments. In addition, cardiac A_2A_-ARs might act via the non-canonical pathway of β-arrestin, via α-actinin, via the Arf nucleotide site opener/cytohesin-2, ubiquitin-specific processing protease, translin-associated protein-X and neuronal calcium-binding protein 2.

The A_2A_-AR gene was first cloned from mice and rats (Libert et al., 1989; Chern et al., 1992). Researchers have generated and studied at least three strains of knockout (KO) mice and two lines of mice with a constitutive cardiac overexpression of A_2A_-AR, as well as one line of mice with an inducible cardiac overexpression of A_2A_-AR (Ledent et al., 1997; Chen et al., 1999; Xiao et al., 2006; Boknik et al., 2018, Boknik et al., 2019; see Table 1). The A_2A_-AR gene contains two exons (Fredholm et al., 2000) and is located on human chromosome 22 (MacCollin et al., 1994). The gene can be alternatively spliced, which could explain the different responses to adenosine exhibited by patients (Haskó and Pacher 2008; Soma et al., 1998). The A_2A_-AR belong to the class of G protein-coupled heptahelical receptors (Figure 1; Fredholm et al., 2001, Fredholm et al., 2011). Mutations to dissect the ligand binding sites and the sequences involved in the signal transduction of the receptor have been extensively studied and reviewed (Fredholm et al., 2001, Fredholm et al., 2011). Polymorphism are known (Deckert et al., 1996; Zhai et al., 2015; Nardin et al., 2018). The human receptor contains 410 amino acids, while the mouse receptor has 409 amino acids; the apparent molecular weight is 45–55 kDa on gel electrophoresis (Fredholm et al., 2001; McIntosh and Lasley 2012). The homology of mouse and human A_2A_-AR is about 90% (Fredholm et al., 2001).

**TABLE 1:** A_2A_-adenosine receptor knock out (KO) and cardiac overexpression mice.

Type			References
KO			Ledent et al. (1997)
KO			Chen et al. (1999)
KO			Xiao et al. (2006)
Flox			Reutershan et al. (2007), Shen et al. (2008), Bastia et al. (2005)
Overexpression	Constitutive	Cardiac specific	Boknik et al. (2018), Boknik et al. (2019), Chan et al. (2008)
Overexpression	Inducible	Cardiac specific	Hamad et al. (2010)

The three-dimensional structure of A_2A_-AR has been studied using crystallization. The X-ray structures of mutated human A_2A_-AR bound to the following agonists have been reported: adenosine or NECA (see Table 2; Lebon et al., 2012), CGS21680 (Lebon et al., 2015), UK-432097 (Xu et al., 2011), an A_2A_-AR agonist and a G protein mimetic (Carpenter et al., 2016), and A_2A_-AR antagonists (Table 3; Jaakola et al., 2008; Doré et al., 2011). The often used agonist CGS21680 (Table 2) binds to transmembrane regions 2 and 7 (Lebon et al., 2015). Nuclear magnetic resonance spectroscopy was used to understand the coupling of A_2A_-AR to G protein signal transduction. This has been addressed with a special focus on the linking role of Asp52^2.50^ (Massink et al., 2015; Eddy et al., 2018). Several human promoters of the A_2A_-AR gene have been characterized (Haskó and Pacher 2008; St Hilaire et al., 2009). In part, these promoters are thought to explain the upregulation and downregulation of the receptors under stressful conditions, such as ischemia.

**TABLE 2:** Agonists at A_2A_-adenosine receptors.

Agonist name	Ki nM		
Adenosine	310		Fredholm et al. (2011)
CGS 21680	27		Fredholm et al. (2011)
	1,570 canine		Glover et al., 2001
UK-432097	4		Fredholm et al. (2011)
ATL-146e (Apadenoson)	0.5		Fredholm et al. (2011)
	44 canine		Glover et al. (2001)
MRE 0094 (sonedenoson)	ND		Fredholm et al. (2011)
(CV-3146) regadenoson	290		Fredholm et al. (2011)
BVT 115959	Not disclosed		https://clinicaltrials.gov/search/intervention = BVT.115959
NECA	9.7		Fredholm et al. (2011)
HE-NECA	2.2		Darbousset et al. (2014)
(WRC-0470) binodenoson	290		Fredholm et al. (2011)
WRC-0090			Shryock and Belardinelli (1997)
WRC-0013			Shryock and Belardinelli (1997)
UK371104	Anti lg 7.7		Jacobson et al. (2019)
GW328267X	Anti lg 8.63		Jacobson et al. (2019)
9 ATL 313 (evodenoson)	Anti lg 9.15		Jacobson et al. (2019)
LASSBio-294	9,500		da Silva et al. (2017)
PSB-15826	14.8 human recombinant		De Filippo et al. (2016)
PSB-12404		IC50: 67	Fuentes et al. (2018)
PSB 16301		IC50: 5.5	Fuentes et al. (2018)
ATL-193	45.8 canine		Glover et al. (2001)
PSB-033	44		El-Tayeb et al. (2011)
CV 1808	190		Cunha et al. (1996)
AMP597	56		Clark et al. (2000)
UK-432094	4.8		Xu et al. (2011)
LUF5834	28		Beukers et al. (2004)

IC50: in functional assays in µM.

**TABLE 3:** A_2A_-adenosine receptor antagonists.

Antagonist aname	Ki nM	Indications	References
Theophylline	1710		Fredholm et al. (2011)
Caffeine	9,560–23,400		Fredholm et al. (2011)
Istradefylline	2–91	M. Parkinson	Fredholm et al. (2011), Cacciari et al. (2018)
Tozadenant	5	M. Parkinson	Cacciari et al. (2018)
ZM 241385	0.8		Cacciari et al. (2018)
MSX-2	5–8.0		Fredholm et al. (2011), Cacciari et al. (2018)
SCH 58261	1.1–5		Fredholm et al. (2011), Cacciari et al. (2018)
SCH 442416	0.048–4.1		Fredholm et al. (2011), Cacciari et al. (2018)
Preladenant	0.9–1.1	M. Parkinson	Fredholm et al. (2011), Cacciari et al. (2018)
Vipadenant	1.3	M. Parkinson	Cacciari et al. (2018)
ST 1535	6.6–11		Fredholm et al. (2011), Cacciari et al. (2018)
ST 4206	9		Cacciari et al. (2018)
CGS 15943	1.2		Fredholm et al. (2011)
CSC	54		Fredholm et al. (2011)
V2006	1.3		Fredholm et al. (2011)

The range of Ki values is probably due to species differences and small difference in methodology.

### Signal Transduction

In general, signal transduction (Figure 1; Table 4 of the A_2A_-AR involves binding to stimulatory guanosine triphosphate-binding proteins (Gs) in peripheral tissues (Kull et al., 2000; Fenton and Dobson 2007), phosphatidylinositol 3-kinase (Schulte and Fredholm 2000; Boucher et al., 2004), and an increase in the amplitude of Ca^2+^ transients (Woodiwiss et al., 1999) or the action on actinin (Burgueño et al., 2003). However, the positive inotropic effect of A_2A_-AR activation is not solely dependent on increases of Ca^2+^ in the cytosol of cardiomyocytes. In rat ventricular cardiomyocytes, activation has also been found to depend on Ca^2+^ independent mechanisms (i.e., an increased Ca^2+^ sensitivity of the myofilaments) (Woodiwiss et al., 1999). Due to the various physiological functions performed by different regions of the heart, it can be hypothesized that the expression and the signal transduction mechanisms of A_2A_-AR could differ between cardiomyocytes in several regions. However, this hypothesis should be tested. Researchers have found that the stimulation of A_2A_-AR increased cyclic-3′-5′-adenosine-monophophate (cAMP) levels, stimulated cAMP-dependent protein kinase (PKA) and phosphorylated cAMP response element-binding protein (CREB) (Németh et al., 2003), and activated non-canonical protein kinase B (AKT), extracellular signal-regulated kinases (ERK), protein kinase C (PKC) (De Ponti et al., 2007; Fredholm et al., 2007), and p38 signaling in skin cells (Perez-Aso et al., 2014).

**TABLE 4:** A_2A_-adenosine receptor: Signal transduction.

Signal	Species/cell type		References
PP2A		Inhibition	Tikh et al. (2008)
PP1		Translocation and activation	Revan et al. (1996)
	Dermal fibroblasts		
Thrombin induced ERK phosphorylation		Inhibits	Hirano et al. (1996)
Gs			Kull et al. (2000), Fenton and Dobson (2007)
PI kinase			Schulte and Fredholm (2000), Boucher et al. (2004)
Ca^2+^	Increased		Woodiwiss et al. (1999)
Actinin			Burgueño et al. (2003)
Ca sensitivity	Increased		Woodiwiss et al. (1999)
Phospho erk	Increased	Mouse heart	Ribé et al. (2008)
Phospho-p-38	Increased	Mouse heart	Ribé et al. (2008)
Phospho-JNK	Increased	Mouse heart	Ribé et al. (2008)
Free radicals	Reduced	Mouse heart	Ribé et al. (2008)
Ca^2+^ sparks	Increased	Atrial human cardiomyocytes	Llach et al. (2011)
CREB phosphorylation	Increased	Leukocytes	Koshiba et al. (1999), review: Rabadi and Lee (2015)
Free radicals	Reduced	Neutrophils	Jordan et al. (1997)
Epac	Activated	Dermal fibroblasts	Perez-Aso et al. (2013)

Interestingly, A_2A_-AR can also exert inhibitory effects in signal transduction; for example, it can inhibit thrombin-induced ERK1/2 phosphorylation (Hirano et al., 1996). It is thought that the receptor probably increases the levels of phosphorylated ERK1/2, p38 mitogen-activated protein kinase (MAPK), and c-Jun N-terminal kinases (JNK) in mouse hearts; higher levels of these proteins have been found in the hearts of wild-type (WT) mice than in the hearts of A_2A_-AR KO mice (Ribé et al., 2008). This finding was thought to explain why the production of free radicals was lower in the hearts of WT mice than in the hearts of A_2A_-AR KO mice (Ribé et al., 2008). The stimulation of the A_2A_ receptor increased the cAMP levels, Ca^2+^ transients, and phospholamban and troponin I phosphorylation states in transgenic (TG) mice with a cardiac overexpression of A_2A_-AR, but not in WT mice (Boknik et al., 2018, Boknik et al., 2019). From these data, we can assume that more Ca^2+^ can be released from the sarcoplasmic reticulum (SR) because activation of the A_2A_-AR via PKA increases the phosphorylation state of the cardiac ryanodine receptor (RYR2), which opens the RYR2 (Figure 1; Llach et al., 2011).

Activation of the A_2A_-AR can also activate protein phosphatases, namely PP1, and can lead to the translocation of the PP1 activity from the soluble fraction to the particulate fraction (Revan et al., 1996). This would lead to the dephosphorylation of target proteins. Researchers also found that the activation of A_2A_-AR in mouse hearts inhibits the activity of PP2A in the myocardial particulate fraction, although this effect was not present in preparations from A_2A_-AR KO mice (Tikh et al., 2008). Interestingly, the researchers found that the stimulation of the A_1_-AR increased PP2A activity to a higher extent than the stimulation of the A_2A_-AR in WT mice (Tikh et al., 2008).

### Interactions of the A_2A_-AR With Other Proteins

**Figure 2:**
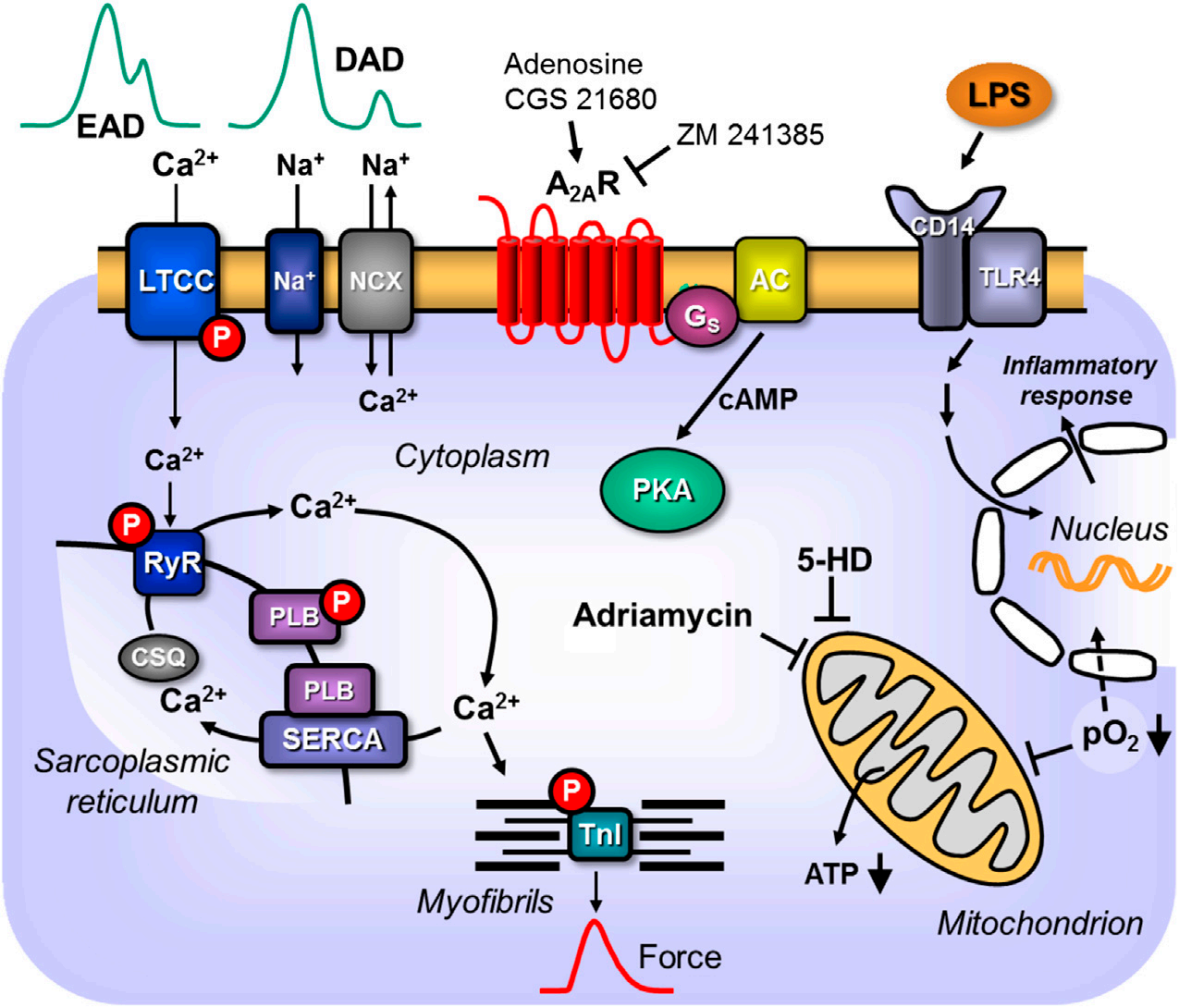
Scheme: Putative pathophysiological role(s) of cardiac A_2A_-ARs. A_2A_-ARs can enhance LPS via receptors in the sarcolemmal alters nuclear gene transcription and putative detrimental proteins are made that impair cardiac contractility. Hypoxia and ischemia impair respiration and thus ATP formation in mitochondria or activate directly hypoxia dependent transcription factors. The mitochondrial process is attenuated by compounds like 5-hydroxy-deanoate (5-HD). Adriamycin impairs mitochondrial function. Hypertension can lead to hypertrophy which alters cardiac gene expression in detrimental ways leading to arrhythmias and heart failure. Altered expression of sarcolemmal ion channels and stimulation of A_2A_-ARs can lead to supraventricular or ventricular arrhythmias by alteration of Ca^2+^ homeostasis.

Studies have reported an interaction between A_2A_-AR and A_1_-AR (Chan et al., 2008, Table 5). In brief, the cardioprotective effect from the stimulation of the A_1_-AR was absent in the isolated hearts from A_2A_-AR KO mice after reperfusion following 30 min of global ischemia (Zhan et al., 2011). Another study found that the A_2A_-AR in SH-SY5Y neuroblastoma cells formed heterodimers with cannabinoid CB1 receptors (Carriba et al., 2007). A_2A_-AR can also interact with the Arf nucleotide site opener/cytohesin-2, ubiquitin-specific processing protease, translin-associated protein-X, and neuronal calcium-binding protein 2 (Ciruela et al., 2010). The A_2A_-AR can form homodimers, homomultimers, and heterodimers with A_1_-AR, dopamine D_2_ receptors, and A_2A_-AR (Moriyama and Sitkovsky, 2010; Ferré et al., 2014; Bonaventura et al., 2015; Navarro et al., 2016). Moreover, homodimers or homomultimers of A_2A_-AR can also be formed (Canals et al., 2004). Interestingly, the dimeric form and not the monomeric form of the A_2A_-AR is thought to be the functional form (Burgueño et al., 2003). The A_2A_-AR can also form dimers and multimers with metabotropic glutamate receptor 5, *N*-Methyl-D-Aspartate (NMDA) receptors and cannabinoid receptors (Headrick et al., 2013; Table 5; Figure 2). Functionally, the A_1_-AR, and possibly the A_3_-AR, can inhibit the relaxation in mouse aortic rings mediated by A_2A_-AR (Talukder et al., 2002; Tawfik et al., 2005). The A_2A_-AR can also form complexes with D_2_ dopamine receptors (Borroto-Escuela et al., 2011).

**TABLE 5:** A_2A_-adenosine receptor interactions.

Receptor			References
A1	Function mouse heart	Cardioprotection inotropy	Tikh et al. (2006), Zhan et al. (2011), Methner et al. (2010), Urmaliya et al. (2010), Chan et al. (2008)
A1	Function rat heart		Norton et al. (1999)
A1		Heterodimer formation	Review: Ferré et al. (2014)
A2A		Homodimer, homomultimer formation	Canals et al. (2004), Burgueño et al. (2003), Ferré et al. (2014)
A2B		Cardioprotection	Moriyama and Sitkovsky (2010)
A3	Mouse aortic rings	Function inhibited	Tawfik et al. (2005), Talukder et al. (2002)
CB1	Neuroblastoma cells	Heterodimer formation	Carriba et al. (2007)
D2		Heterodimer formation, heterotetramer formation	Navarro et al. (2016), Bonaventura et al. (2015)
			Ferré et al. (2014)
D2-mGlu5			Cabello et al. (2009)
D2-CB1			Navarro et al. (2008)
D2-calmodulin			Navarro et al. (2009)
D3			Torvinen et al. (2005)
Arf nucleotide site opener			Ciruela et al. (2010)
Ubiquiting-specific processing protease			Ciruela et al. (2010)
Translin-associated protein-X			Ciruela et al. (2010)
Neuronal calcium-binding protein 2			Ciruela et al. (2010)

An interaction between the A_1_-AR and the A_2A_-AR occurs on a functional level in the heart. Stimulation of the A_1_-AR reduces the positive inotropic effect (i.e., an increase in the force of contraction) of isoproterenol, which is a β-adrenoceptor agonist. This well-known effect was attenuated in perfused rat hearts through the additional stimulation of the A_2A_-AR (Norton et al., 1999), as well as in the perfused hearts from WT mice; however, the effect was absent in the hearts of A_2A_-AR KO mice (Tikh et al., 2006). Similarly, isoproterenol increased Ca^2+^ transients in electrically stimulated rat ventricular cardiomyocytes; this effect was attenuated through the activation of A_1_-AR and the addition of A_2A_-AR antagonists (Norton et al., 1999). The constitutive overexpression of the A_1_-AR in the heart led to cardiac dilatation in mice, which was not seen in mice coexpressing A_2A_ and A_1_ receptors; this might suggest there is a beneficial interaction between these receptors that is determined by the different effects of expression of the sarcoplasmic reticulum Ca^2+^ ATPase (SERCA) (Chan et al., 2008).

### Expression

**Figure 3:**
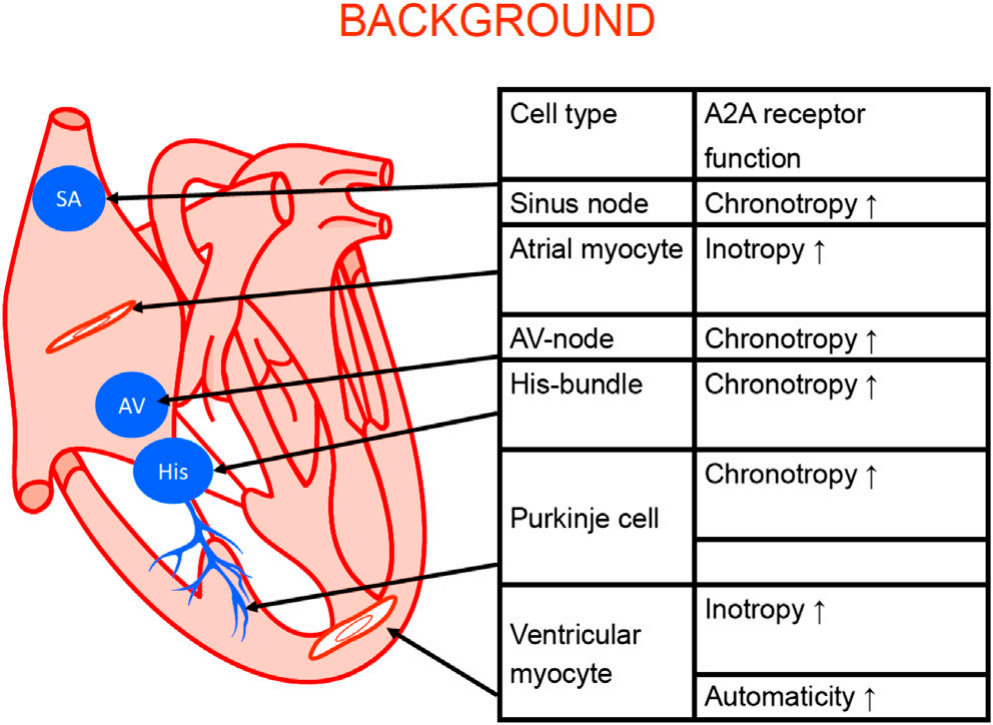
Schematic cardiac conducting system and regional adenosine receptor expression in the heart (modified from Stein et al., 1998). The anatomical localization of the sinus node (SA), the AV node (AV) the bundle of His (His). The Purkinje fibers (Purkinje) and the work generating muscle cells in the atrium and the ventricle are depicted. In these structures A_2A_-ARs were functionally and/or biochemically detected. Their stimulation can explain the functional consequence listed in the adjoining table.

There are several types of cardiomyocytes in the heart: atrial cardiomyocytes form the main bulk of atrial muscle; ventricular cardiomyocytes; cardiomyocytes that form the path of the system that propagates depolarization from the sinus node, specialized cells in the atrium (Bachmann bundles), the sinus node (SA) node, Tawara branches, and Purkinje bundles (Figure 3). Alterations in this pathway are expected to be of clinical relevance because they can lead to various cardiac arrhythmias. Alterations in the expression of A_2A_-AR might be relevant for both primary arrhythmias due to inborn errors and secondary arrhythmias from ischemia, hypertrophy, drug treatment, or aging. However, more work on this topic needs to be undertaken.

In terms of the expression and cellular heterogeneity of A_2A_-AR in the heart, these receptors are known to be present and functional in blood cells that are continuously transported to and from the heart by the circulatory system (Table 6). A_2A_-AR comprise various types of leukocytes, macrophages, mast cells (Marquardt et al., 1994), neutrophils (Fredholm et al., 1996), thrombocytes, and erythrocytes. In histological studies, such as RNA detection using single cell polymerase chain reaction (PCR) or *in situ* hybridization, it is sometimes possible to clearly identify the receptor in different cell types; in studies using antibodies, the specificity can be poor. However, when using cardiac homogenates, the A_2A_-AR can be measured in all cell types and one might assume that the signal mainly arises from the cardiomyocytes but the signal might arise also from other cell types in the heart. For example, in one study, all P1 receptors, including A_2A_-AR, were detectable in the heart using reverse transcription and PCR (see Table 6). Adult rat ventricular cardiomyocytes, as well as adult mouse and porcine ventricular cardiomyocytes, contained A_2A_-AR mRNA and protein (Xu et al., 1996; Marala and Mustafa 1998; Kilpatrick et al., 2002; Chandrasekera et al., 2010). Moreover, A_2A_-AR mRNA and protein were detected in human atrial preparations using Western blotting (Hove-Madsen et al., 2006; Llach et al., 2011); according to its histochemistry, A_2A_-AR were located with the cytoskeletal-associated protein α-actinin at the Z-line of the sarcomere in the atrial specimens (Hove-Madsen et al., 2006). A_2A_-AR have also been detected on endothelial cardiac cells (Hein et al., 1999) and vascular smooth muscle cells (Teng et al., 2008).

**TABLE 6:** A_2A_-adenosine receptor: Tissue Distribution in heart and blood.

Tissue	Species/cell type	References
1. Cardiomyocytes		
1.1	Adult rat	Xu et al. (1996); Kilpatrick et al. (2002)
1.2	Pig	Marala and Mustafa (1998)
1.3	Human	Hove-Madsen et al. (2006); Marala and Mustafa (1998); Llach et al. (2011)
1.4	Mouse	Chandrasekera et al. (2010), Morrison et al. (2006)
2. Blood cells		
2.1	Platelets	Amisten et al. (2008)
2.2	Mast cells	Review: Gao and Jacobson (2017)
2.3	Macrophages	Review: Haskó and Pacher (2012)
2.4	Neutrophils	Fredholm et al. (1996)
2.5	Erythrocytes	Kamata et al. (2008)
3. Vascular smooth muscle cells		Teng et al. (2008)
4. Coronary endothelial cells		Hein et al. (1999), Olanrewaju and Mustafa (2000)
5. Lymphocytes		Review: Brown et al. (2008)
6. Basophils		Review: Brown et al. (2008)
7. Fibroblasts	Rat heart	Chen et al. (2004), Epperson et al. (2009)

### Altered A_2A_-AR Levels

The level of A_2A_-AR in tissues can be altered by various stimuli. Changes in these levels may have relevance to cardiac diseases. For example, carbon monoxide increases the expression of A_2A_-AR in macrophages (Haschemi et al., 2007). Similarly, when lipopolysaccharide (LPS) was used to induce inflammation, the expression of A_2A_-AR increased in murine and human macrophages and epithelium cells via the (nuclear factor kappa-light-chain-enhancer of activated B cells (NF-κB) pathway (Murphree et al., 2005; Morello et al., 2006; Haskó and Pacher 2008). Hypoxia via hypoxia-inducible factor like HIF2α also increased the expression of A_2A_-AR in pulmonary endothelial cells (Ahmad et al., 2009). Pharmacological treatment can also increase A_2A_-AR: caffeine increas the A_2A_-AR density in for instance platelets (Varani et al., 1999). Likewise, diazepam treatment can increase the function of A_2A_-AR stimulation in rat pulmonary arteries (Ujfalusi et al., 1999). D_2_-dopamine receptor stimulation could increase the function (cAMP production) in neuroblastoma cells (Vortherms and Watt, 2004).

The expression of A_2A_-AR can also be diminished. For instance, increased cAMP levels reduced the expression of A_2A_-AR in cell cultures (Headrick et al., 2013). A_2A_-AR agonists can lead to desensitization (see Table 7), as seen with many other G protein-coupled receptors, possibly through binding the receptor to α-actinin and receptor internalization (Chern et al., 1993; Svenningsson et al., 1995, Svenningsson et al., 1999); this effect does not occur with antagonists (Halldner et al., 2000). In DDT1 MF-2 and PC12 cells, short-term (less than 30 min) treatment with an agonist reduced the subsequent induced increases in cAMP levels without any loss of A_2A_-AR on the cell surface (Ramkumar et al., 1991; Chern et al., 1993; Klaasse et al., 2008). Another study reported a functional desensitization after 2 h of treatment with NECA, which reduced the vasorelaxation of porcine coronary arteries after a second exposure of NECA (Conti et al., 1997). In cell cultures mutational analysis revealed there were different phosphorylation sites on the A_2A_-AR (Palmer and Stiles, 1997). In human monocytoid THP-1 cells, tumor necrosis factor alpha (TnF-α) inhibited the agonist-induced desensitization of A_2A_-AR by preventing the translocation of G-protein-coupled receptor kinase 2 (GRK2 to the plasma membranes (Khoa et al., 2006). The desensitization of A_2A_-AR seems to involve arrestin 2 and 3 (Burgueño et al., 2003). It is thought that the internalization of A_2A_-AR involves its C-terminus and its interaction with α-actinin (Burgueño et al., 2003).

**TABLE 7:** A_2A_-adenosine receptor sensitization and desensitization.

Agonist/Intervention	Tissue	Read out		References
NECA	Relaxation of coronary bovine arteries	Force	Desensitization	Conti et al. (1997)
NECA	DD1 MF cells	cAMP	Desensitization	Ramkumar et al. (1991)
NECA	PC12 cells	cAMP	Desensitization	Chern et al. (1993)
Hypoxia	Pulmonary endothelial cells	A2A-AR mRNA	Sensitization	Ahmad et al. (2009)
Caffeine	Platelets	A2A-AR radio-ligand binding	Sensitization	Varani et al. (1999)
LPS				
Diazepam	Rat pulmonary arteries	Relaxation		Ujfalusi et al. (1999)
D2 receptor stimulation	Neuroblastoma cells	cAMP		Vortherms and Watts (2004)

### Role *of* A_2A_-AR in Cardiac Disease

#### Arrhythmia

In TG mice, the genetic overexpression of A_2A_-AR (Figures 2, 3; Table 1) in the heart makes them much more susceptible than WT mice to negative inotropic effects and to the arrhythmogenic effects of intraperitoneally injected adriamycin (Hamad et al., 2010). This suggests that A_2A_-AR have a detrimental reaction to stressors, such as adriamycin; adriamycin is an anti-cancer drug that can cause heart failure and arrhythmias in some patients in a dose- and time-dependent manner. Compared with WT mice, the A_2A_-AR in TG mice might be more susceptible to adriamycin-induced arrhythmias because they express less connexin 43, which is a protein that is important for myocardial conduction (Hamad et al., 2010). Interestingly, when A_2A_-AR overexpression was induced after treatment with adriamycin, the mortality of the TG mice was lower than that of the WT mice (Hamad et al., 2010). One study reported that mice with a constitutive cardiac specific overexpression of A_2A_-AR had an increased basal heart rate (Chan et al., 2008). In patients with atrial fibrillation, the expression of A_2A_-AR was increased (Csóka et al., 2010). Stimulation of the A_2A_-AR in isolated human cardiomyocytes increases Ca^2+^ sparks by increasing the Ca2+ current through the sodium–calcium exchanger (I_NaCa_), which can lead to cellular depolarization, initiate afterdepolarizations, and cause cardiac arrhythmias (Figure 2; Llach et al., 2011). A_2A_-AR have also been expressed in human atrial preparations, which may lead to alterations in the frequency of spontaneous Ca^2+^ release (Hove-Madsen et al., 2006). Alternatively, the action of adenosine can induce bradycardia and subsequently lead to atrial fibrillation (Isa-Param et al., 2006). Ischemia can also induce increases in adenosine, which can lead to arrhythmias (Bertolet et al., 1997).

Interestingly, one study found that atrial dilation and atrial fibrillation were accompanied by an increase in A_2A_-AR mRNA and protein levels, which was conceivably due to an increase in RyR2 phosphorylation (Llach et al., 2011). This may alter the flow of Ca^2+^ through the sarcolemmal sodium–calcium ion exchanger (NCX) and lead to arrhythmias (Figure 2; Llach et al., 2011). The study found that the stimulation of the A_2A_-AR induced altered Ca^2+^ sparks in isolated human atrial myocytes (Llach et al., 2011). Moreover, CGS21680 enhanced currents through the NCX in isolated atrial cardiomyocytes from patients with atrial fibrillation, but not in samples from patients in sinus rhythm (Llach et al., 2011). Interestingly, endogenous adenosine is able to stimulate NCX in atrial cardiomyocytes from patients with atrial fibrillation (Llach et al., 2011).

It has been suggested that the stimulation of A_2A_-AR may increase the Ca^2+^ content of the SR and the stimulation of the NCX might increase Ca^2+^ inflow in cells. The increased levels of Ca^2+^ in the SR may result in an increased release of Ca^2+^ from the SR, which can lead to delayed afterdepolarization and, thus, to atrial arrhythmias, such as atrial fibrillation (Figure 2). There is evidence that the stimulation of A_2A_-AR mediates vasodilation, which may lead to tachycardia by reducing blood pressure. The decrease in blood pressure leads to reflective tachycardia through the stimulation of the baroreceptor, which stimulates the sympathomimetic outflow from the central nervous system. In living rats, the stimulation of the A_2A_-AR leads to tachycardia by the direct activation of the sympathetic tone and by the release of noradrenaline, which stimulates the β-adrenoceptors on the sinus node (Dhalla et al., 2006). Using telemetric electrocardiograms, we confirmed the positive chronotropic effect of A_2A_-AR expression alone and its stimulation by a selective A_2A_-AR agonist in living animals (Boknik et al., 2019). It is relevant that we could detect an enhanced incidence of arrhythmias in living animals after stimulation of the A_2A_-AR because it may indicate that the proarrhythmic effect of A_2A_-AR expression is so strong that vagal or other neural compensatory mechanisms cannot overcome it (Boknik et al., 2019). We predict that the same might apply in humans. There is experimental evidence that the positive chronotropic effect of A_2A_-AR stimulation by regadenoson was caused by the direct stimulation of the sympathetic nervous system in rats (Woodiwiss et al., 1999). Another study showed that the stimulation of A_2A_-AR in isolated human atrial myocytes promoted irregularities in Ca^2+^ transients, such as spontaneous calcium ion waves (Csóka et al., 2010). Spontaneous Ca^2+^ release has been reported to initiate atrial fibrillation in human atrial myocytes (Monahan et al., 2000; Jiang et al., 2011; Llach et al., 2011). Future studies should determine whether the increase in A_2a_-AR is the cause or the result of atrial fibrillation in humans.

#### Ischemia and Hypoxia

In general, A_2A_-AR exert a protective role in the heart. For instance, the A_2A_-AR can protect the brain against ischemia (Jiang et al., 2011; Fronz et al., 2014; Melani et al., 2014). However, contradictory results have been reported. For instance, one study found that the stimulation of A_2A_-AR produced detrimental effects in the brain of A_2A_-AR KO mice, while an antagonist was associated with beneficial effects in the brain in theses mice (Phillis, 1995; Chen et al., 1999; Chen et al., 2007). Similar effects were noted in the kidney, which could be partly explained by the fact that A_2A_-AR mediate vasodilation and anti-inflammatory effects by reducing the production of cytokine and chemokine in leukocytes, including macrophages, lymphocytes, and neutrophils, in the kidney (Rabadi and Lee, 2015). The mechanism may involve cAMP-dependent phosphorylation of CREB and subsequent alterations in gene transcription (Rabadi and Lee, 2015). The activation of A_2A_-AR in regulatory T lymphocytes (Koshiba et al., 1999) also plays a part in renal protection (review: Rabadi and Lee, 2015; Lasley, 2018). However, the stimulation of the A_2A_-AR in mice with cecal ligation had detrimental effects, as seen in that model of chronic inflammation and sepsis (Haskó and Pacher, 2008). Thus, depending on the method of disease generation, as well as the acute or chronic nature of inflammation, A_2A_-AR play two contrasting roles in the heart. As previously mentioned, reperfusion leads to cardiac dysfunction if ischemia is prolonged, which is partially due to non-cardiomyocytes, such as neutrophils. The activation of A_2A_-AR diminished neutrophil adherence to endothelial cells and decreased the production of superoxide anions; this might partly mediate reperfusion injury in the mammalian heart (Jordan et al., 1997). However, in rabbits, the stimulation of A_2A_-AR could reduce cardiac ischemia-induced arrhythmia (Schreieck and Richardt, 1999).

In the present context, A_2A_-AR could protect the myocardium of adult rats with an coronary occlusion (Lozza et al., 1997; Ke et al., 2015). A_2A_-AR agonists also protect the heart function against septic injury (Thiel et al., 1998; Tofovic et al., 2001; Braun-Dullaeus et al., 2003; Reutershan et al., 2007). However, what is the role of endogenous adenosine in sepsis? In A_2A_-AR KO mice, sepsis was more pronounced than in WT mice (Reichelt et al., 2013; Ashton et al., 2017) or unaltered (Reutershan et al., 2007). Therefore, the role of A_2A_-AR in sepsis might be quite subtle; age and gender might also be a factor, as older male KO mice exhibited a poor prognosis (Ashton et al., 2017). A_2A_-AR also protect against postconditioning (Dhalla et al., 2006; Morrison et al., 2007; review; McIntosh and Lasley 2012) and preconditioning (Button et al., 2005; Yang et al., 2005) and ischemia and reperfusion *in vivo* rats (Kis et al., 2003; Ke et al., 2015). However, some studies of preconditioning showed that the stimulation of A_2A_-AR prior to ischemia did not provide any protection against a decrease in force (reviewed in McIntosh and Lasley, 2012). Activation of A_2A_-AR in postconditioning might be of special therapeutic utility for patients with coronary heart disease. Reperfusion of coronary arteries in patients by balloon dilation can sometimes lead to arrhythmias or a reduction in the force of contraction to levels lower than those before occlusion. These detrimental complications of reperfusion could be attenuated in the clinic by giving an A_2A_-AR-agonist intravenously before reopening the occluded artery. For instance, rats in an *in vivo* ischemia model were given A_2A_-AR agonists, such as CGS21680, which was beneficial in preventing biochemical signs of autophagy (Ke et al., 2015). Cardiac specific overexpression of A_2A_-AR in mice, increased the sustaining of pressure after reperfusion, possibly by altering the electrical properties of cardiomyocytes and these beneficial effects were absent when A_2A_-AR antagonists were used supporting the studies on a beneficial effect of A_2A_-AR stimulation prior to ischemia and reperfusion. This beneficial effect translates to a longer time to contracture during ischemia in these hearts. The beneficial effect might be due effects on the mitochondria Boknik et al., 2018.

A lack of beneficial effects from the activation of A_2A_-AR on cardiac preconditioning has also been reported (Lasley and Mentzer, 1992; Thornton et al., 1992; Yao and Gross, 1993; Lasley et al., 2007). This result might be due to the use of different species or methods, such as the type and dose of agonist used, and the timing of the agonist application.

It is possible that more than one type of cell is involved in the mechanism of cardioprotection. Lymphocytes, neutrophils, mast cells, basophils, dendritic cells, monocytes, epithelial cells, endothelial cells, and macrophages contain A_2A_-AR (Revan et al., 1996; Jordan et al., 1997; Germack and Dickenson, 2005; Rork et al., 2008; Csóka et al., 2012; Burnstock and Boeynaems, 2014). A_2A_-AR are also present on platelets; activation of these receptors inhibits platelet aggregation (review: Burnstock 2015). It has been suggested that the A_2A_-AR contribute to the functional cardioprotective action of the A_1_-AR (Methner et al., 2010; Urmaliya et al., 2010; Zhan et al., 2011). A beneficial interaction between the A_2A_-AR and the A_2B_-AR has been described in the heart (Xi et al., 2009). The protective mechanism of the A_2A_-AR seems to involve actions on the mitochondria of the heart in rats (Ke et al., 2015). In pulmonary endothelial cells, hypoxia via HIF2α not only increases the density of the A_2A_-AR mRNA and protein, but also generates more adenosine through the induction of adenosine-producing enzymes (Ahmad et al., 2009). Moreover, the overexpression of A_2A_-AR in human lung microvascular endothelial cells led to an increase in cell proliferation and promoted increased angiogenesis (Ahmad et al., 2009). An increased expression of A_2A_-AR in tumors was noted in patients with lung cancer compared with healthy lung regions from the same patients (Ahmad et al., 2009). In hypoxia, the uptake of adenosine into cells is diminished, which leads to high extracellular concentrations of adenosine that activate the A_2A_-AR (Eltzschig et al., 2005; Löffler et al., 2007; Morote-Garcia et al., 2009). Low concentrations of adenosine are expected to bind to and activate the high-affinity A_1_-AR; in a pathophysiological context, further increases in adenosine activate the A_2A_-AR because of their low affinity for adenosine. However, in functional cAMP production in cells, A_1_-AR and A_2_-ARbshow the same affinity for adenosine (Fredholm, 2014). Under basal physiological conditions, adenosine concentrations range between 30 and 200 nM, which are sufficient to activate both A_1_-AR and A_2A_-AR (Fredholm, 2014). Very high levels of adenosine (1 µM adenosine) were reported after platelet aggregation (Fredholm, 2014), ischemia, and necrotic cell death (Fredholm, 2014). The A_2A_-AR can lead to the dilation of coronary arteries and might be deleterious in patients with Morbus Parkinson (Fredholm, 2014). Clinically, adenosine and its precursor ATP are useful for stopping supraventricular arrhythmias. Therefore, the actions of adenosine in mammalian hearts are of clinical relevance and merit further investigation.

Whereas A_1_-AR and A_3_-AR protect the heart when activated before ischemia, stimulation of A_2A_-AR can protect a rat heart at the beginning of a reperfusion injury (Jordan et al., 1997; Cargnoni et al., 1999; Yang et al., 2006; Kuno et al., 2008; Headrick and Lasley, 2009; Xi et al., 2009; Methner et al., 2010). Overexpression of A_2A_-AR protects against cardiac damage because the enzymatic activity of AST, which is a marker for the inability of the sarcolemma to contain ingredients within the cell, only increased in the WT mice and not in the TG mice (Boknik et al., 2018). In all probability, this protection was mediated by the A_2A_-AR because the protective effect in the TG mice was abolished by applying the A_2A_-AR antagonist ZM 241385 (Boknik et al., 2018). The protective effect may involve the mitochondria, as the phosphorylation state of pAKT was increased to a higher extent during reperfusion in the TG mice than in the WT mice (Boknik et al., 2018, 2019).

It might be speculated that if A_2A_-AR are beneficial in reperfusion, one might try to increase the levels of A_2A_-AR in the heart by injection of adenovirus containing the cDNA for the A_2A_-AR intravenously or even in the coronary arteries. In this way it may be possible to increase the A_2A_-AR in leukocytes but also in coronary endothelial cells. The amount of the adenovirus should probably not be so elevated as also the increase A_2A_-AR in cardiomyocytes: though this might improve cardioprotection (Boknik et al., 2018), there is the danger to induce sinus tachycardia or other arrhythmias (Boknik et al., 2019), known for all cAMP-increasing agents.

#### Heart Failure

There is a debate about whether A_2A_-AR are functional (i.e., increase cAMP and contractility) in the mammalian heart; the effect of A_2A_-AR may also be species- or method-dependent. Studies have reported a lack of effect in the rat (Shryock et al., 1993), guinea pig (Behnke et al., 1990; Boknik et al., 1997), and rabbit (Kilpatrick et al., 2002). However, other studies have reported a functional response in mice (Morrison et al., 2006) and rats (Monahan et al., 2000). It is important to note that A_2A_-AR protein levels have been detected in human hearts (Marala and Mustafa, 1998). Work on isolated and perfused A_2A_-AR KO (i.e., constitutive deletion) mouse hearts clearly established that the A_2A_-AR agonist CGS21680 was selective; <1 µM CGS21680 increased contractility in WT mouse hearts, but not in hearts from A_2A_-AR KO mice (Morrison et al., 2002). Furthermore, we noted a functional role of A_2A_-AR stimulation in isolated paced right atrial preparations from diseased human hearts (Boknik et al., 2018). However, under basal conditions in which no CGS21680 was given, there was no difference in the contractility of WT and KO mouse hearts (Ashton et al., 2017; Morrison et al., 2002). The stimulation of the A_2A_-AR produced a positive inotropic effect (Table 8; Woodiwiss et al., 1999; Monahan et al., 2000; Chan et al., 2008; Dobson et al., 2008; Tikh et al., 2008), which increased in the presence of additional A_1_-AR blockade DPCPX (Fredholm et al., 2011 for selectivity of DPCPX). One study noted that the stimulation of A_2A_-AR had no positive inotropic effect (Kilpatrick et al., 2002). Some studies reported that the A_2A_-AR increased in patients with heart failure (Stein et al., 1998), while one study found that A_2A_-AR mRNA level decreased in Japanese patients with heart failure (Asakura et al., 2007). The plasma adenosine concentration increases in human heart failure (Funaya et al., 1997). In neutrophils and T cells, the expression of A_2A_-AR is regulated by miR-214, miR15, and miR 16 (Heyn et al., 2012). Other studies have reported on a constitutive cardiac specific overexpression (Chan et al., 2008) or an inducible overexpression (Hamad et al., 2010) of human A_2A_-AR in a mouse heart. One group used their model to study the *in vivo* functional interaction of A_1_-coexpression with A_2A_-AR (Chan et al., 2008). A_2A_-AR have a protective role in pressure overload from aortic banding (Hamad et al., 2012) and against cardiomyopathy from chronic adriamycin treatment (Hamad et al., 2010).

**TABLE 8:** Species- and region-dependent cardiac effects of stimulation of cardiac A_2A_-adenosine receptors.

Species/tissue			A2A-AR agonist used
Human atrium	Boknik et al. (2018)	PIE	CGS 21680
Human ventricle	ND		
Rat cardiomyocytes	Woodiwiss et al. (1999)	PIE	CGS 21680
Perfused rat ventricle	Monahan et al. (2000)	PIE	Adenosine
Rat ventricular cardiomyocytes	Shryock et al. (1993)	No PIE	WRC 0090
			WRC 0013
Guinea pig ventricular cardiomyocytes	Shryock et al. (1993)	No PIE	WRC 0090
			WRC 0013
Rabbit ventricular cardiomyocytes	Shryock et al. (1993)	No PIE	WRC 0090
			WRC 0013
Guinea pig atrium	Böhm et al. (1986)	No PIE	NECA
Guinea pig ventricle	Isolated cardiomyocytes: Behnke et al. (1990), Boknik et al. (1997)	No PIE	NECA
		No PIE	CGS 21680
Mouse atrium	Wild type mice: left atria, Boknik et al. (2018), Boknik et al. (2019)	No PIE	No PCE
		CGS 21680	CGS 21680
Mouse atrium	A2A receptor overexpressing mice atria: Boknik et al. (2018), Boknik et al. (2019)	PIE	PCE
		CGS 21680	CGS 21680
Mouse ventricle wild type	Tikh et al. (2008), Boknik et al. (2018), Boknik et al. (2019)	PIE	CGS 21680
			CGS 21680
Mouse ventricular cardiomyocytes wild type	Dobson et al. (2008)	PIE	CGS 21680
Mouse ventricle	A2A receptor overexpressing mice: Boknik et al. (2018)	PIE	PCE
		CGS 21680	CGS 21680
Rabbit ventricle	Kilpatrick et al. (2002)	No PIE	CGS 21680

ND, not done; No, no inotropic effect; PCE, positive chronotropic effect; PIE, increase in contractility.

It might be speculated that physiological alterations of the A_2A_-AR occur in myocardial ischemia; for instance, changes could happen during infarction and stenting of a vessel. Therefore, the receptors may present a target for the pharmacological treatment of cardiac arrhythmias. However, more detailed studies are needed.

Following ischemia in the brain, there was an increased expression of A_2A_-AR (Trincavelli et al., 2008). The beneficial effects of A_2A_-AR activation have been reported in autoimmune diseases, such as colitis, rheumatoid arthritis, and hepatitis, as well as after mechanical trauma to the nervous system (Choukèr et al., 2008; Di Paola et al., 2010; Mazzon et al., 2011; Paterniti et al., 2011). Polymorphisms of the A_2A_-AR have been correlated with human chronic heart failure (Zhai et al., 2015).

It is questionable to stimulate endogenous (or by application of an adenovirus containing the cDNA for the A_2A_-AR), as the A_2A_-AR will increase cAMP and cAMP increase can lead to arrhythmias. Another caveat is in order: to the best of our knowledge, it has not been reported that A_2A_-AR agonist can increase force of contraction in the human ventricle, though this is usually taken for granted (Table 8).

### Clinical Relevance of A_2A_-AR Agonists and Antagonists

A_2A_-AR activation is used in clinics to assess the vasodilatory functions of coronary arteries during nuclear magnetic studies. Adenosine is also used to treat supraventricular arrhythmias. Some of the adverse effects of adenosine include flushing and hypotension; these effects have been attributed to the action of vasodilatory A_2A_-AR (review: Headrick et al., 2013). The A_2A_-AR antagonists istradefylline and tozadenant are new compounds that have been used to treat patients with Morbus Parkinson (review: Chen et al., 2013) or sickle cell disease (Chen et al., 2013).

Many people drink coffee or tea that contains caffeine and theophylline at levels that can block A_1_-, A_2A_-, A_2B_- and A_3_-AR (Fredholm et al., 1999; Chen et al., 2013). Thus, caffeine might interfere with agonists, but might potentiate antagonists. Clinical studies have found that caffeine was beneficial in patients with Morbus Parkinson (Chen et al., 2013).

A_2A_-AR agonists and antibodies have also been clinically applied (Table 9; Cargnoni et al., 1999; Cerqueira et al., 2008; Palani et al., 2011; Molina et al., 2016). For instance, the A_2A_-AR agonists ATL 146e and MRE-0094 have been investigated to promote wound healing in patients with chronic neuropathic diabetic foot ulcers; they have also been used to treat arthopathies; lung diseases, such as COPD; hepatic ischemic diseases; renal ischemic diseases; inflammatory bowel disease; and ischemic brain diseases (Haskó and Pacher, 2008). One possible drawback to using these agonists is that they stimulate the A_2A_-AR on vascular smooth muscle cells, which would lead to vasodilation and a subsequent decrease in blood pressure (Hein et al., 1999; Haskó et al., 2006). Thus, these agonists may be used as antihypertensive agents, but should not be used to treat foot ulcers. After chronic stimulation of A_2A_-AR, the downregulation of a homologous receptor might occur for many G protein-coupled receptors, such as the 5-HT_4_-serotonin receptor (Gergs et al., 2017). If the stimulation of an immune receptor is intended, then an A_2A_-AR antagonist for cardiac disease might be tried. For instance, A_2A_-AR antagonists have been used at the clinic for the treatment of patients with Morbus Parkinson. Moreover, theophylline, which is used to increase mucociliary clearance in patients with COPD, and dipyridamole, which inhibits adenosine transporters on cell surfaces, increase adenosine levels near A_2A_-AR. Adenosine and its metabolite inosine can be transported through cell membranes by bidirectional and concentrative transporters (Podgorska et al., 2005). One of the side effects associated with the use of adenosine to stop paroxysmal supraventricular arrhythmias involves bronchoconstriction by the stimulation of the bronchostrictory A_2A_-AR (Chen et al., 2013). A_2A_-AR agonists, such as regadenoson, are clinically used to detect latent ischemia in patients (Cerqueira et al., 2008). Other experimental agonists include CGS21680, UK-432097, and BVT115959 (Jacobson and Müller, 2016). Therefore, activation of A_2A_-AR could lead to arrhythmias in patients that have high expression levels of A_2A_-AR in the heart.

**TABLE 9:** Diseases suggested to involve A_2A_-adenosine receptors (review: Burnstock, 2017).

Disease	Therapy	References
M. Alzheimer	Agonist	Cunha (2016)
M. Parkinson	Antagonist	Jenner (2014)
Amyotrophic lateral sclerosis	Antagonist	Volonté et al. (2016)
Brain injury	Agonist	Dai and Zhou (2011)
Schizoprenia	Agonist	Matos et al. (2015)
Depression	Antagonist	Yamada et al. (2014)
Addiction (e.g. cocain)	Antagonist	Pintsuk et al. (2016)
Cardiac ischemia/reperfusion injury	Agonist	Ke et al. (2015)
Atrial fibrillation	Antagonist	Molina et al. (2016)
Atherosclerosis	Agonist	Reiss and Cronstein (2012)
Coronary would healing	Agonist	Du et al. (2015)
Thrombosis	Agonist	Hofer et al. (2013)
Asthma	Agonist	Wang et al. (2018)
COPD	Agonist	Basu et al. (2017)
Acute lung injury	Agonist	Friebe et al. (2014)
Cystic fibrosis	Agonist	Esther et al. (2013)
Lung cancer	Antagonist	Mediavilla-Varela et al. (2013)
Pleural inflammation	Agonist	da Rocha Lapa et al. (2012)
Rhinosinusitis	Agonist	Hua et al. (2013)
Diabetes	Antagonist	Ibrahim et al. (2011)
Obesity	Antagonist	De Oliveira Moreira, et al. (2017)
Acute renal injury	Agonist	Vincent and Okusa (2015)
Diabetic nephropathy	Agonist	Persson et al. (2015)
Liver fibrosis	Antagonist	Ahsan and Mehal (2014)
Liver cirrhosis	Agonist	Choukèr et al. (2008)
Psoriasis	Agonist	Merighi et al. (2017)
Scleroderma	Antagonist	Chan and Cronstein (2010)
Skin wound healing	Agonist	Shaikh and Cronstein (2016)
Myasthenia gravis	Agonist	Oliveira et al. (2015)
Osteoarthritis	Agonist	Corciulo et al. (2017)
Rheumatoid arthritis	Agonist	Mazzon et al. (2011)

### Ongoing Clinical Studies

There is an ongoing study using oral AB928, a novel dual A_2a_R/A_2b_R antagonist (Seitz et al., 2019) to treat prostate cancer by inhibiting AR mediated cell proliferation (University of California, United States: clinical trials.gov identifier: NCT04381832). There is another trial on prevention of injury of ischemia and reperfusion using regadenoson which is an A2AR agonist: in this case the ischemic injury in the lung blood vessels in lung transplantation will be tried to treat using intravenously applied regadenoson (University of Maryland, United States: clinical trials.gov NCT04521569). A similar trial is ongoing in Toronto, Canada (clinical trials.gov identifier: NCT04521569) Another trial is mainly intended to used expression levels of A2A-AR as a prognostic factor in cardiovascular disease. More specifically this study is evaluating the discriminating capacities of A2A adenosine receptors expression on the surface of circulating lymphocytes for the detection of coronary artery disease in patients hospitalized for surgery of the aorta and/or arteries of the lower limbs (Marseille, France: clinical trials.gov identifier NCT04640844).

### Open Questions

The roles of various canonical and non-canonical pathways of A_2A_-R signal transduction merit further investigation. Biased agonists might offer new therapeutic options. The exact roles of A_2A_-AR in chronic heart failure, ischemia, and reperfusion, as well as in cardiac protection against myocardial infarction and arrhythmias need to be more carefully studied and translated into clinical settings. It remains unclear whether A_2A_-AR agonists or antagonists might be useful. It is conceivable that interactions with other receptors are important in therapeutic drug development. It is unknown whether an upregulation or downregulation by receptor ligands, adenoviral approaches, or antisense RNA approaches would be clinically useful. Many of these questions will be answered in the near future and this progress will be observed with great interest. It is hoped that developments will be used to benefit patients.

## Summary

Over the years a consent has emerged that A_2A_-AR are present and functional in the mammalian heart, more importantly in the human heart. Evidence has accumulated that

The A_2A_-AR is important for coronary flow, which is relevant under clinical conditions and used in to assess e.g. the coronary reserve in patients with coronary heart disease. A_2A_-AR might be relevant for force generation in the human heart and for the genesis of arrhythmias. Hence, A_2A_-AR agonist and antagonists are clinically used. A_2A_-AR agonist mainly for diagnostic purposes (assessment of coronary reserve) and might be used in the future as positive inotropic agents. A_2A_-AR antagonist might become useful as novel options in some subtypes of heart failure.

## Author Contributions

JN initiated the project. BH, NZ, and UG added own topics and aspects. UG, PB, and JN finalized the figures and body text. All authors read and approved submission of this version.

## Conflict of Interest

The authors declare that the research was conducted in the absence of any commercial or financial relationships that could be construed as a potential conflict of interest.
